# Pollen Protein: Lipid Macronutrient Ratios May Guide Broad Patterns of Bee Species Floral Preferences

**DOI:** 10.3390/insects11020132

**Published:** 2020-02-18

**Authors:** Anthony D. Vaudo, John F. Tooker, Harland M. Patch, David J. Biddinger, Michael Coccia, Makaylee K. Crone, Mark Fiely, Jacob S. Francis, Heather M. Hines, Mackenzie Hodges, Stephanie W. Jackson, Denis Michez, Junpeng Mu, Laura Russo, Maliheh Safari, Erin D. Treanore, Maryse Vanderplanck, Eric Yip, Anne S. Leonard, Christina M. Grozinger

**Affiliations:** 1 Department of Biology, University of Nevada Reno, Reno, NV 89557, USA; jacob.franci@gmail.com (J.S.F.); anneleonard@unr.edu (A.S.L.); 2 Department of Entomology, Center for Pollinator Research, The Pennsylvania State University, University Park, PA 16802, USA; tooker@psu.edu (J.F.T.); hmpatch@psu.edu (H.M.P.); hmh19@psu.edu (H.M.H.); ezt5142@psu.edu (E.D.T.); ecy7@cornell.edu (E.Y.); cmgrozinger@psu.edu (C.M.G.); 3 Fruit Research and Extension Center, Pennsylvania State University, Biglerville, PA 17307, USA; djb134@psu.edu; 4 Department of Psychiatry, University of California San Francisco, San Francisco, CA 94143, USA; michael.coccia@ucsf.edu; 5 Intercollege Graduate Degree Program in Ecology, Huck Institute of the Life Sciences, The Pennsylvania State University, University Park, PA 16802, USA; mkc206@psu.edu; 6 Ernst Conservation Seeds, Inc., Meadville, PA 16335, USA; hortpath@ernstseed.com (M.F.); swunderley@gmail.com (S.W.J.); 7 Museum of Life and Science, Durham, NC 27704, USA; mhodges5961@gmail.com; 8 Laboratory of Zoology, University of Mons, Mons B-7000, Belgium; denis.michez@umons.ac.be (D.M.); maryse.vanderplanck@umons.ac.be (M.V.); 9 Ecological Security and Protection Key Laboratory of Sichuan Province, Mianyang Normal University, Mianyang 621000, China; mujunpeng@gmail.com; 10 Department of Entomology and Plant Pathology, University of Tennessee, Knoxville, TN 37996, USA; lrusso@utk.edu; 11 Department of Biochemistry and Biophysics, University of California San Francisco, San Francisco, CA 94158, USA; maliheh.safari@ucsf.edu

**Keywords:** bee health, floral rewards, nutritional ecology, pollen foraging behavior, pollination ecology, plant–pollinator interactions

## Abstract

Pollinator nutritional ecology provides insights into plant–pollinator interactions, coevolution, and the restoration of declining pollinator populations. Bees obtain their protein and lipid nutrient intake from pollen, which is essential for larval growth and development as well as adult health and reproduction. Our previous research revealed that pollen protein to lipid ratios (P:L) shape bumble bee foraging preferences among pollen host-plant species, and these preferred ratios link to bumble bee colony health and fitness. Yet, we are still in the early stages of integrating data on P:L ratios across plant and bee species. Here, using a standard laboratory protocol, we present over 80 plant species’ protein and lipid concentrations and P:L values, and we evaluate the P:L ratios of pollen collected by three bee species. We discuss the general phylogenetic, phenotypic, behavioral, and ecological trends observed in these P:L ratios that may drive plant–pollinator interactions; we also present future research questions to further strengthen the field of pollination nutritional ecology. This dataset provides a foundation for researchers studying the nutritional drivers of plant–pollinator interactions as well as for stakeholders developing planting schemes to best support pollinators.

## 1. Introduction

Declines in the abundance and diversity of flowering plant species generate nutritional stressors for bees, driving population declines of wild and managed bee species across the world [1,2,3,4,5]. Thus, there is increasing interest in defining the nutritional needs of bee species and using this information to optimize planting schemes that support bee communities. To this end, nutritional ecology theory, which focuses on the mechanisms of foraging for multiple nutrients [6,7], can offer a new perspective on plant–pollinator interactions that are useful for conservation. Bees integrate and balance multiple macronutrient resources when making foraging decisions that have consequences on their health and fitness [8,9,10]. Understanding the complex connections between the nutritional resources that plants provide and the nutritional needs of bees reveals insights into bee–flower symbioses and coevolution, and ultimately the assembly and stability of plant–pollinator communities. Here, we (1) review research leading to the understanding that protein to lipid (P:L) ratios of pollen can drive bee foraging behavior and health, (2) present pollen P:L ratio data for over 80 plant and three bee species, (3) discuss observed patterns in P:L ratios that may influence plant–pollinator interactions, and (4) present future research questions that might guide the growing field of pollination nutritional ecology.

Bees (Hymenoptera: Anthophila) rely on floral resources for development, survival, and reproduction [11]. Nectar provides foraging bees with their main source of carbohydrates [12], while pollen is the main source of proteins and lipids for bees [13]. Pollen must be consumed by larvae for proper development (or consumed by nurse honey bees to produce royal jelly), while consumption by female adults is essential for reproduction [14,15] and is linked to health and disease resistance [16]. Plant species vary widely in pollen quality including protein concentration [17,18,19,20], amino acid composition [21], lipid concentration [18,19,20,22], fatty acid profiles [23], sterol composition [24,25], and secondary metabolites reviewed in [26]. Because differences in pollen identity, nutrient composition, and diversity affect bee health, polylectic and oligolectic species alike must choose between host plants to obtain appropriate pollen nutrition for themselves and offspring.

Since pollen is the primary source of protein and essential amino acids for nearly all bee species, historically, analyses of pollen quality and its link to bee health and foraging behavior were based on protein content alone [27,28,29,30,31,32,33,34,35,36,37,38,39,40], yet results varied. In laboratory colonies, increasing protein and amino acid concentrations in pollen diets improves bumble bee colony growth [29,38], improves honey bee health [16,40], and increases brood size of *Lasioglossum zephyrum* [27]. Bumble bees visit flowering plant species with higher levels of pollen protein [33,34] collect pollen that is higher in protein than honey bees [37], and prefer pure over diluted pollen diets [35,36]. They can also taste and discriminate different groups of amino acids dissolved in water [41]. However, evidence does not support the hypothesis that honey bees choose pollen of higher protein concentrations [31,32]. Furthermore, *Lasioglossum zephyrum* does not prefer pollen diets higher in protein [27]. Although the protein content of pollen is clearly important for bee health, the choice of diet based on increasing protein concentrations varies between bee taxa, and thus, there may be other pollen nutritional factors related to bee pollen preferences.

Recently, lipids have emerged as a second major influence on bee foraging and health. In particular, the lipid-rich external pollenkitt can act as a discriminative and phagostimulant for bees [41,42], and bumble bees can perceive and learn pollens spiked with fatty acids [43]. Moreover, low omega 6:3 fatty acid ratios are linked to increased learning and memory performance in honey bees [23,44], while increased oleic acid in diet increases memory and survival in bumble bees [45]. Higher pollen sterol levels have been repeatedly linked to bumble bee colony health and fitness [24,25]. Dietary needs based on the composition of lipid and sterol nutrients are likely to be species-specific; different bee taxa show characteristic body-lipid composition likely derived from their pollen diet [46], and lipid preference may guide host–plant choice for specialists and generalists alike [47]. The overconsumption of diets too high in lipid content can be detrimental to bumble bee health and possibly lead to their avoidance [8,43]. Therefore, the specific composition of pollen lipids may also be a determining factor leading to host–plant choice and bee–flower interactions. 

Because bees undoubtedly forage for pollen to meet both their protein and lipid needs, both macronutrients should be considered together as possible drivers of bee foraging preferences. Our research on bumble bees considered pollen as a multidimensional nutritional resource, using a conceptual framework from the field of nutritional ecology, the Geometric Framework (GF, [48] and reviewed in [6,7]). This body of theory has been widely used to understand how animals forage adaptively for multiple nutrients. This framework suggests that all animals have optimal concentrations and ratios of nutrients, termed “intake targets”, for homeostasis, growth and development, and reproduction. Typically, food sources differ in concentrations and ratios of nutrients and often do not match the intake target of most species. Therefore, foraging animals need to sense food quality and adjust their foraging to mix consumption from multiple food sources, regulating their dietary intake to reach their targets. 

As pollen is the main source of protein and lipids for bees, and these values differ considerably between plant species, bees may multidimensionally assess pollen quality and balance pollen nutritional intake via ratios of proteins and lipids. We tested this hypothesis by studying the foraging behavior and health of *Bombus impatiens* based on protein to lipid ratios (P:L). In flight arenas of mixed plant species, we found that pollen foraging rates increased exponentially to higher P:L ratios among plant species (maximum of 5:1 P:L) [18,49]. To test if pollen nutrition alone drove this choice, we presented *B. impatiens* workers paired choices of pollen from different flowering plant species or nutritionally modified pollen diets. In this experiment, they consistently preferred comparatively higher P:L ratios [18]. We found similar evidence of nutrient foraging by collecting pollen from free-flying *B. impatiens* colonies deployed in different habitats. Notably, all colonies regardless of habitat type collected similar diets, averaging approximately 4:1 P:L across all pollen loads, which was similar to the target ratio established among pollen diets previously [9]. Finally, using synthetic diets, we showed that both *B. impatiens* and *B. terrestris* workers are capable of simultaneously regulating lipid and protein intake [8]. Thus, we concluded that bumble bee pollen foraging preferences were driven by high P:L ratios.

Recent studies have demonstrated further associations between bee foraging preferences and health and pollen P:L ratios. For example, entire bumble bee colonies regulate protein and lipids to similar ratios as we predicted previously [10]. We found that the P:L of different pollens used in agricultural cover-cropping systems have differential effects on *B. impatiens* colony ovary activation and wax production [20]; we also found that P:L ratios and the protein content of an invasive plant species may be more attractive to the bee community (including solitary bees) than surrounding native plants, presenting a possible mechanism of invasive competitive ecology [19]. While there is mounting evidence that P:L ratios are important for driving plant–pollinator interactions, these studies have focused on a small number of plant or bee species (mostly *Bombus* species). Likewise, only a few studies have conducted wide assessments of pollen nutritional quality across plant species, but most of these have only analyzed one nutrient or only a handful of species (e.g., [17,18,19,21,24,29,50]). Since analytical methodologies have differed between studies, they have yielded varying species-specific nutrient composition values [17,18,21,51,52,53]. A recent meta-analysis of pollen nutritional values was the first to consolidate data across studies using different analytical techniques to analyze multiple pollen nutrients and reveal broad phylogenetic trends [54]. This study suggested that there are generally higher protein and P:L ratios found in bee-pollinated versus wind-pollinated plants (although pollen collected directly from bees had not been included). 

Our interest has been to characterize and predict specific bee species’ P:L nutritional intake based on pollen nutritional values of specific plant species. Therefore, we analyzed P:L ratios for pollen for over 80 mostly bee pollinated plant species and three bee species (*Apis mellifera*, *Bombus impatiens,* and *Osmia cornifrons*). Pollen was collected by the co-authors during the course of their research, which spans a range of fields including bee health, bee–flower interactions, invasive ecology, plant physiology, and habitat restoration. By using a standardized methodology to analyze pollen nutrition across these plant and bee species, we hope to provide a framework for directly comparing pollen nutrition from plant and bee sources. Therefore, researchers can determine the nutritional space occupied by different plant species and ultimately understand how different bee species may differentially forage within that space to reach their pollen intake targets.

## 2. Materials and Methods

We collected pollen from 82 plant species and three bee species (*Apis mellifera, Bombus impatiens*, and *Osmia cornifrons*). Pollen collection involved multiple methods, which we classified as “fresh” (collected directly from dehisced anthers on flowers), “anther” (collecting anthers from flowers in the field and collecting pollen in the lab), “mass” (vacuuming pollen from flowers), or “bee collected” (from *A. mellifera* pollen traps, *B. impatiens* corbiculae, or *O. cornifrons* nest provisions; Table S2). Each plant species pollen was pooled from hundreds of flowers needed to collect sufficient pollen for analysis (> 10 mg). We further classified bee-collected pollen as “bee” if it was a mixed pollen sample collected from free-foraging bees (useful for evaluating bees’ P:L intake targets), or “plant” if it was monofloral pollen collected by honey bees from pollen traps (see Supplementary Protocol S1 for details on pollen collection methodology). To prevent nutrient degradation, we immediately stored all pollen at −20 °C or −80 °C until analysis (in the field, we placed pollen on ice and then stored it in the freezer upon return to the lab). Depending on the experiment, we either dried pollen at 36 °C for 24 h prior to analysis, lyophilized for 3 h (*O. cornifrons*), or analyzed the pollen fresh (see Table S2). For each pollen sample, three replicates of approximately 1 mg each were used for protein, and three replicates of approximately 1 mg each were used for lipid analyses. We used the same pollen protein and lipid analytical protocol detailed previously [9,18,19,20,50] (see Supplementary Protocol S1 for a step-based protocol).

**Figure 1:**
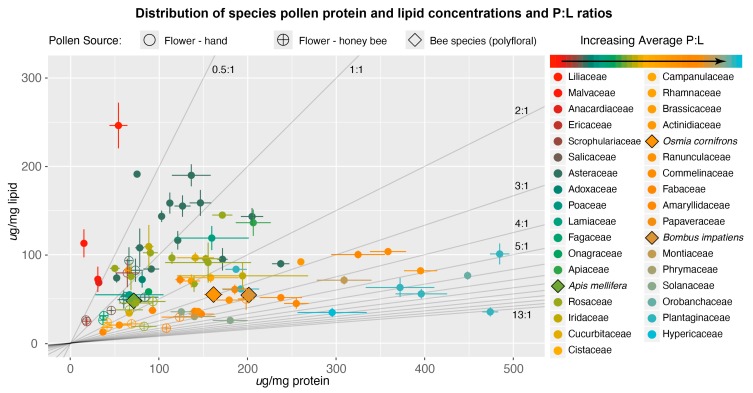
Plant and bee species mean ± SE protein and lipid concentrations (ug/mg) in two-dimensional nutritional space. Each marker represents a separate plant (circle) or bee species (diamond) colored along a gradient from low to high pollen protein to lipid (P:L) ratios by plant family (and bee species italicized). See Figure 2 for the average P:L values per family and Table S2 for individual species protein, lipid, and P:L values. Filled circles represent plants measured from pollen collected directly from flowers, open circles are monofloral pollen species collected by honey bees, and filled diamonds with black outlines are the average of mixed pollen loads of bee species. Gray lines represent nutritional rails, which are labeled by P:L values.

**Figure 2:**
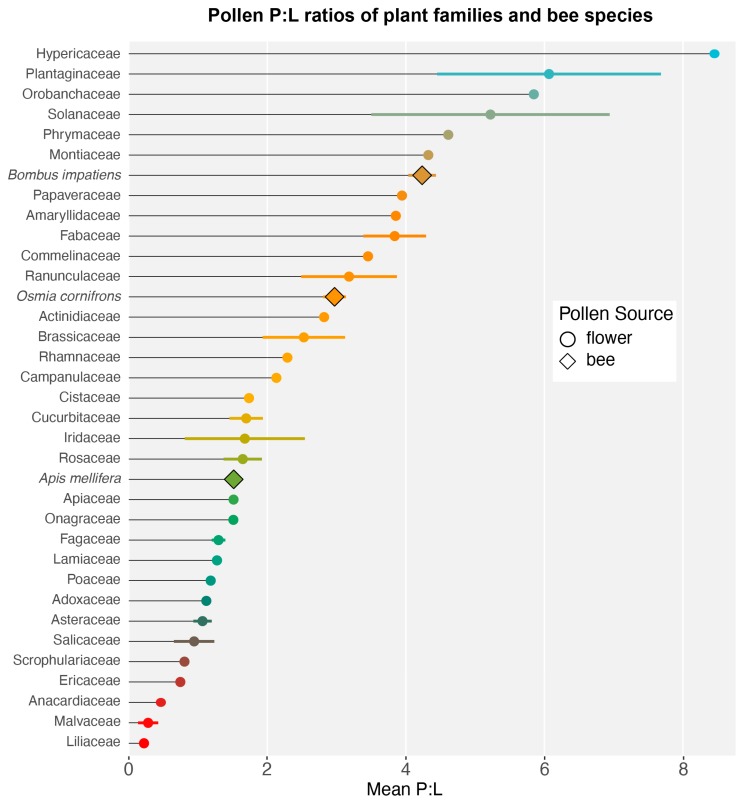
Plant family and bee species mean ± SE (where available) P:L values. Circles represent plant families, and diamonds represent bee species.

For each plant and bee species, we calculated the average protein concentration (mean ± SE ug/mg) and average lipid concentration (mean ± SE ug/mg; Table S2) of the three subsamples of pollen (some species included multiple treatments/populations: *Carduus nutans*, *Chamaecrista fasciculata*, *Cistus spp.*, *Helianthus annuus, Salix caprea*, *Solidago altissima*). Because honey bees add nectar to pollen, which changes the nutrient concentrations per weight [55], we treated any repeated plant species that we collected from honey bees as separate samples. We determined the pollen P:L values of plant species by dividing the average protein by average lipid concentration; for bee species, we averaged the P:L values across all pollen loads (Table S2). To visualize the nutritional distribution of plant species, we plotted mean ± SE protein and lipid concentrations (Figure 1). We plotted nutrient P:L “rails” [48], which represent a constant nutritional ratio that exists alongside a range of specific nutrient concentrations (Figure 1). To visualize taxonomic trends in P:L values, we determined the mean ± SE P:L (when available) value for each plant family (Figure 2). We included bee species average P:L values to visualize where and how they differ in nutritional space (Figure 1 and Figure 2).

## 3. Results and Discussion

### 3.1. Nutritional Content of Pollen

Pollen species and bee-nutrition values occupy a wide range of concentrations and ratios (Figure 1, Table S2). Previous analyses of pollen nutrients ranged from approximately 2% to 60% protein and 1% to 20% lipids [17,22], or 7% to 50.8% protein and 0.48% to 17.6% lipid [54]. Our data are similar; from fresh collected pollen, species concentrations ranged from 1.5% to 48.4% protein and 1.2% to 24.6% lipid. The resulting range of P:L ratios across our mostly bee-pollinated plant species was 0.13–13.3 P:L. Neither nutrient was exclusively associated with pollen P:L values, with protein content positively and lipid content negatively correlated to P:L value (generalized linear model: *X*^2^ = 113, *P* < 0.0001). Notably, the majority of the plant species and families we analyzed fall within P:L ratios < 3:1, exhibiting a median of approximately 1.7:1 and mean of approximately 2.5:1 P:L (Figure 1 and Figure 2). These results support the hypothesis that there are physiological, ecological, and phylogenetic limits on pollen nutritional values [17,54]. Perhaps the requirements of producing and depositing other pollen components (exine, intine, sugars, water, which can add up to 50% of pollen weight [22]) limit plants from increasing concentrations of protein and lipid without increasing all other components. The interactive costs of producing various nutrients may lead to constraints on nutrient concentrations available to pollinators (i.e., the tradeoff between pollen functions of reproduction and reward [17,54]).

Our analysis suggests that the P:L ratio is biologically and ecologically informative, and that it is also a robust metric across different collection methods. For instance, as mentioned above, corbiculate bees add nectar to pollen, reducing the absolute concentrations of protein and lipid nutrients per weight. Nevertheless, our analysis identified the P:L ratios of this bee-collected pollen as being nearly the same as the pollen collected directly from flowers (see Table S2). This is likely because both protein and lipid concentrations are being equally affected; thus, the ratios remain stable. Similarly, drying pollen prior to analysis, rather than analyzing it completely fresh, will concentrate nutrients and change nutritional content per unit mass. Yet, our results suggest that P:L ratios were again comparatively similar (Table S2), which should allow for consistent comparisons among species. Our analysis of both honey bee collected monofloral pollen and dried pollen fit within the general family level P:L ratios obtained from fresh pollen (e.g., average Asteraceae P:L of approximately 1:1 or high P:L values of Fabaceae; Figure 1, Table S2). 

A recent meta-analysis of pollen nutrition revealed the evolutionary function of nutrient rewards by emphasizing the degree to which pollen nutrient content was conserved phylogenetically among plants mainly pollinated by bees [54]. Although our data revealed some similar family-level trends among P:L values, they are not completely congruent. Overall, the average P:L ratios among plant species and families in the meta-analysis tended to be much higher than our results. This difference could relate to the different chemical analytical protocols used across different studies compiled in the meta-analysis, while we used the same protocol to analyze all the species we studied. These differences illustrate a need in future studies to utilize standard methods to make comparisons across pollen nutritional datasets.

### 3.2. P:L Trends in Bee–Flower Interactions

Here, we evaluate the P:L ratios of pollen samples collected by three bee species: *Apis mellifera*, *Bombus impatiens*, and *Osmia cornifrons*. As data for *A. mellifera* and *O. cornifrons* were not collected for systematic analysis, we acknowledge that these trends, while intriguing, require further study with a robust design. It is also likely that bee species’ preferences and nutritional targets may be species, caste, and life-stage specific and not driven simply by higher or increasing nutrient concentrations or ratios [6,10,56]. Furthermore, bee foraging for particular pollen nutritional values may be attenuated by the limits of landscape availability. Nonetheless, in the hopes of encouraging future research in this area, we discuss the trends in the P:L ratios of pollen that we measured in relation to the foraging behavior of the three focal bee species.

Our research with bumble bees indicated that in controlled settings (i.e., laboratory and flight arena), their preferred P:L ratios of pollen ranged from 5:1 to 10:1 [8,18]. In the field, *B. impatiens* collected from a wide variety of plant species, yet averaged a ratio of 4:1 [9]. This indicates that bumble bee P:L preferences are above the median and mean of all the plant species and families we analyzed (Figure 1 and Figure 2). Although generalist foragers, *B. impatiens* prioritize high P:L ratios in pollen and require a specific subset of available plant species; thus, the overall abundance of preferred nutrients in the landscape may drive colony growth [9,57]. For instance, we observed high abundances of bumble bees and pollen rewarding flowers with high P:L ratios (e.g., *Lupine*, *Pedicularis,* and *Penstemon*) in protected alpine meadows (Vaudo and Leonard in prep; Table S2). The nutritional drivers of large patterns of bumble bee decline could be linked to the loss of specialized floral resources (e.g., Fabaceae, Solanaceae, *Penstemon*, etc.) that have pollen with high P:L ratios (Figure 2) [1,2,58]. 

Based on the samples analyzed in this study, honey bees collected pollen between 1:1 and 2:1 P:L, which appears to occupy different nutritional space than pollen collected from bumble bees, which was approximately 4:1 (Figure 1 and Figure 2). As generalists, honey bees collect from many host-plant species, and given that many plant species and families have a relatively low range of P:L ratios, pollen collected by honey bees may reflect this overall distribution of P:L values (Figure 1). Furthermore, to satisfy the demands of colonies with thousands of individuals, honey bees must collect large quantities of pollen. Therefore, honey bees collect from generalist, open floral morphologies such as mass blooming trees (e.g., *Quercus*, *Salix*, *Prunus*) and wildflowers (Asteraceae, *Brassica*) that fall in lower P:L values [59,60]. So, even though honey bees and bumble bees are generalists, in the same habitat, they occupy different nutritional spaces based on the different utilization of floral resources [37] (Figure 1 and (Figure 2). Although honey bees do not necessarily prefer flowers with higher protein values [31,61], they may still be selective for pollen nutrition. The composition of spring pollen has important effects on honey bee health [62], and foragers may be able to balance the colony nutritional intake of amino and fatty acids [63,64]. Thus, we need to further test whether honey bees are selective for nutritional quality or if their intake reflects the local landscape distribution of nutrients.

Contrary to honey bees and bumble bees, *Osmia cornifrons* is a solitary foraging bee with a short flying period. It was introduced to the United States from Japan in 1977 and managed for rosaceous fruit tree pollination [65]. Previous studies of *O. cornifrons* pollen preferences suggest their pollen affinity to Rosaceae and Fabaceae [66,67]. Interestingly, the average of the Rosaceae (1.6 ± 0.3 P:L) and Fabaceae (3.8 ± 0.5P:L) P:L ratios is similar to the average P:L value we found for *O. cornifrons* (approximately 2.9 P:L) presented here (Figure 2). Although not a definitive analysis, perhaps *O. cornifrons* mainly balances its diet between these pollen sources. Other mesolectic and oligolectic bees may also reach their nutrient targets through the ability to forage from fewer and distinct diet sources to regulate their diet [68].

A challenge of foraging bees is to both balance diets for themselves and for their offspring, needing to be sensitive to the pollen protein and lipid requirements of developing larvae [69]. The above three species presented have different larval feeding strategies. *Apis mellifera* nurse bees consume pollen collected by foragers to develop their hypopharyngeal glands (HPG) to produce royal jelly, which is then progressively fed to larvae. The increased diversity and protein content of pollen diets increase HPG development [70,71], but not royal jelly protein quality [72]. As foragers and nurses occupy different tasks, perhaps the low average P:L values described for honey bees above are offset by generalist foraging strategies and nurse bees’ ability to process food quality through royal jelly toward intake targets for larvae. In contrast, although *Bombus* species progressively feed their larvae, adults either directly regurgitate pollen to larval cells or feed pollen into cells through pockets without changing pollen quality [73]. Therefore, both foraging and nurse bumble bees (which only show weak specialization [74]) may be more directly sensitive to larval P:L target requirements. If so, higher selectivity regarding pollen nutrition may guide their foraging. Finally, most solitary bees, such as *O. cornifrons*, mass provision pollen to larvae. Therefore, foraging females are directly responsible for larval food quality and may also be directly sensitive to larval P:L targets, guiding mesolectic or oligolectic foraging patterns [68], and possibly providing an additional explanation as to why larvae cannot survive on non-host pollen [75]. Furthermore, in the subsocial mass provisioning *Ceratina calcarata*, the foraging foundress appears to collect different pollens with different nutritional values (i.e., protein, and quite possibly lipids and sterols) to either rear the non-reproductive worker (“dwarf eldest daughter”) or future reproductives, emphasizing the maternal control and specificity of pollen nutrient collection [76].

Our analysis of bee-pollinated plant species shows trends in pollen P:L ratios that correspond to plant reward strategy (primarily pollen versus nectar rewarding) and pollinator behavior (floral preferences), which generate hypotheses that warrant further systematic analysis. As expected, the primarily wind-pollinated plants such as pistachio (*Pistacia vera*, 0.4 P:L), corn (*Zea mays*, 1.18 P:L), and oak (*Quercus pyrenaica*, 1.2 P:L) have relatively low P:L ratios [54]. In addition, some primarily nectar-rewarding flowers from our dataset also occupy low P:L values, such as Lamiaceae, *Arnica spp.*, and Malvaceae (*Sidalcea* and *Hibiscus*). Interestingly, in both the old and new world, there are bee taxa specialized on Malvaceae pollen (*Tetralonia* and *Diadasia* respectively) that may be equipped to handle these particular low P:L diets [77,78]. On the other hand, specialized floral phenotypes that are handled by behaviorally specialized bees, such as flowers with keels, poricidal anthers, or primarily pollen-rewarding flowers (e.g., Fabaceae, Solanaceae, kiwi, *Pedicularis*, *Penstemon, Tradescantia*) exhibit some of the highest P:L ratios we analyzed. As predicted, bumble bee-pollinated crops, such as kiwi (*Actinidia deliciosa*) and solanaceous crops such as peppers (*Capsicum annuum*), have high P:L ratios. In general, the large family Asteraceae has consistently low P:L ratios (1.06 ± 0.1) [59]. They often produce large amounts of pollen in open arrays of florets and have a wide diversity of pollinator taxa. It is possible that providing large amounts of pollen at relatively low P:L ratios is a pollination strategy; thus, the high abundance of Asteraceae creates environments for many bee species to adapt and specialize to Asteraceae pollen [79,80,81]. Interestingly, we found that an invasive thistle in North America (*Carduus acanthoides*) has high 2.6 P:L ratios relative to other Asteraceae species, which seemed to attract pollinators away from other asters and may facilitate its pollination [19]. A variety of generalist bee-pollinated trees seem to occupy the median P:L range, which may reflect their attractiveness to many pollinators, but some cultivars exhibit quite low P:L ratios in the samples we tested (pear and plum). Additionally, although remaining high, there is substantial difference between the P:L values of Jalapeño and Marengo varieties of *Capsicum annuum* P:L values (Table S2). Crop domestication can reduce nectar and pollen defensive chemistry [82], but the effect of domestication on pollen nutritional chemistry and effect on pollinator attractiveness, pollination services, and health needs further investigation.

Linking bee species to host-plant species based on nutritional factors is obviously still at its nascent stage. At this point, we only have single species representatives for some plant families, and these need to be expanded for confidence in family-level trends. For instance, plants in the family Ericaceae are widely pollinated by bumble bees in the Northern Hemisphere, yet the single species representing this family in our analysis (*Erica spp.*, heath) had very low P:L, which did not fit our predictions for bumble bee pollen collection [24,83]. We also did not systematically sample across plant taxa, principle pollinators, or reward strategies; therefore, more detailed studies are necessary [54]. In addition, we recognize that chemical factors other than P:L ratios influence bee foraging. For example, secondary compounds that certain taxa may be able to detoxify or tolerate could have led to differentiation between bee taxa and host-plant use [26,75,79,84]. Finally, as exemplified in research of *B. impatiens*, comprehensive analyses of pollen choice and nutritional intake for honey bees, *O. cornifrons*, and other bees need to be conducted. 

### 3.3. Applying Pollen P:L in the Future

When defining plant species by their nutritional value, we can more clearly build hypotheses regarding how floral pollen P:L rewards are conserved or vary within and among taxa, and then further deduce how this influences the ways that plants interact with their pollinators. Similarly, we can address the mechanisms that determine bees’ perception of P:L, identify their specific P:L intake targets, and ask how this influences their foraging behavior and fitness. These topics can be integrated to understand community interactions and address conservation. For example, we can study host-plant shifts of bees as their ranges cover areas of plant species turnover, discuss evolutionary shifts of pollinators to new host plants based on diet composition [85], and changes of floral mating systems to other insect or vertebrate pollinators. We can test how variations in the P:L values of pollen species affect bee physiology and health in the lab and field [20]; more sophisticated geometric framework studies [10] are clearly needed for different bee species. Pollen nutrition may also be important for understanding invasive ecology and responses to climate change [19,50,86,87]. For instance, introduced bees may adapt to new geographic regions by shifting to host-plant species with phylogenetically conserved pollen nutrition (Vaudo et al. in prep). We can also study hypotheses correlating nutritional drivers of declines of oligolectic, mesolectic, or polylectic bee species to landscape change through reduced floral diversity [1,2,3,5]. 

Datasets such as that presented here have a variety of uses in bee conservation and habitat restoration. We can select plant species for restoration and augment habitat in wild and agricultural ecosystems by creating nutritionally targeted plant lists [13]. One important insight from an exploratory dataset such as ours is that species diversity alone (i.e., many species within limited families) does not necessarily guarantee nutritional diversity, but selecting nutritionally diverse plant species leads to a diversity of plant families, phenotypes, and morphologies that would attract a diversity of bee species. The same concept can be used to target singular or specialized crop pollinating species. By providing enough nutritionally diverse and phenologically matched resources, we can support target bee species, and the surrounding bee community, by allowing populations to select and regulate their dietary intake [88]. 

### 3.4. Concluding Remarks

Pollen nutrition likely plays an essential role in shaping pollinator communities. Although our research is focused mainly on bees, similar principles can be applied to other pollinators that consume pollen, such as flies, beetles, etc. As we have outlined in this study, we can make many inferences of how pollen P:L links broad patterns of pollinator species host-plant choice to differences in their nutritional intake, contributing to understanding how this may lead to coevolution between plants and pollinators. However, at this early stage of understanding the diversity of bee nutritional needs and plant species nutritional rewards, we have yet to experimentally test these ideas. Therefore, the utility of these types of datasets will increase as we populate them with tested values from a variety of plant and bee species so that we have a more comprehensive understanding of plant–pollinator communities. As we progress in the field, it is important to work toward unified protocols and collaborations so that we may have directly comparable results between studies for larger and more specific phylogenetic, geographic, and temporal inferences.

## Supplementary Materials

The following are available online at https://www.mdpi.com/2075-4450/11/2/132/s1, Protocol S1: Step based protocol for pollen collection and pollen protein and lipid analysis, Table S2: Table of plant and bee species mean and SE protein and lipid concentrations (ug/mg) and P:L ratios. Pollen collectors (and publications where available) are provided, and collection methods are described in Protocol S1.
