# Supplementary material for: Pollen Protein: Lipid Macronutrient Ratios May Guide Broad Patterns of Bee Species Floral Preferences
Source: Insects. 2020 Feb 18;11(2):132. doi: 10.3390/insects11020132 (PMC7074338; doi:10.3390/insects11020132)
Supplement: Supplementary file 1 [file insects-11-00132-s001.zip › Protocol S1 pollen collection and protein lipid analysis.pdf]

Pollen collection

Among the number of techniques to collect and prepare pollen (see Kearns, C. A.; Inouye, D. W. *Techniques for pollination biologists*; University Press of Colorado, USA, 1993), common challenges are 1) efficient harvest of a sufficient mass of pure pollen for an experiment, 2) proper and immediate storage to prevent nutrient degradation, and 3) weighing pollen accurately for analysis. At an absolute minimum, we recommend collecting 10 mg of fresh pollen for analysis, or >15 mg if the pollen will be dried. This amount can require collection of pollen from hundreds to thousands of flowers. The plant and bee species used in the current analysis involved multiple collection methods, which are classified as "fresh", "anther", "mass", or "bee collected" (Table S2). We further classified bee-collected pollen as "bee" if it is a mixed-pollen sample for analyzing bee pollen intake, or "plant" if it is monofloral pollen collected by honey bees. To prevent nutrient degradation, we store placed all pollen -20℃ or -80℃ until analysis (in the field, we first place pollen on ice and store in the freezer immediately upon return to the lab).

Fresh

We collected fresh pollen by preventing pollinator visitation and harvesting directly from flowers. Flowers were either grown in field cages with fine mesh, bagged prior to dehiscence, grown in a greenhouse, or clipped in the field and allowed to dehisce in the lab. Pollen from open flowers was brushed with *synthetic* fine paint brushes (using brushes made from hair may alter protein concentrations), scraped with a razor into glass or plastic vials, or suspended to allow pollen to fall onto paper or foil. For keel shaped flowers (e.g., legumes), we tripped the keel and angled anthers into collection vials. For poricidal anthers and buzz-pollinated flowers (*Chamaecrista*, *Pedicularis, Senna, Solanum*, etc.), we vibrated flowers with electronic toothbrushes, or we placed anthers in eppendorf tubes, and vortexed to release pollen.

Anthers

In the field, we collected non-dehisced anthers from flowers using fine-tipped forceps in the morning. We vortexed dried or fresh anthers in plastic tubes to break anthers and release pollen and create a static environment in which pollen sticks to the sides of the tubes. We then collect pollen by scraping the sides of the tubes with a spatula or scalpel.

Mass

To collect large quantities of pollen from trees, pollen can be harvested from tree flowers using vacuums. The pollen can then be purified pollen by sieving it through fine mesh. However, the pollen will likely not be 100% pure because broken flower parts may still pass through the sieve. We purchased tree pollen species from Antles Pollen Supplies, Inc (Wenatchee, WA) (Table S2).

Bee collected

Pollen trapping from honey-bee hives is an efficient way of collecting large masses of pollen, but it has two limitations. First, one needs to verify pollen identity and its purity via microscopy. Second, because of the nectar that honey bees add to pollen to pack it on to their corbiculae, it can be challenging to accurately measure absolute nutrient concentrations of the source pollen. Fortunately, despite the addition of nectar, P:L ratios should remain stable.

We also analyzed mixed pollen loads from *A. mellifera* (honey bee), *B. impatiens* (bumble bee), and *Osmia cornifrons* (Japanese Orchard Bee). We purchased commercial (Brushy Mountain Bee

Farm, Kelly Beekeeping, Clarkson, KT USA) multifloral honey-bee pollen, and collected other pollens directly from pollen traps on hives. We collected bumble-bee pollen from their legs in the field or pollen traps on their colonies. For *O. cornifrons*, we collected at the end of the growing season pollen from larval provisions in nests. We homogenized honey-bee-collected pollen with mortar and pestle, mixed it well, and randomly sampled it to ensure the nutritional value represented an average of the collected pollen (N=9) [19]. We analyzed bumble bee corbiculate pollen individually (N=~300) [11], or mixed as above. We also analyzed each *O. cornifrons* larval provision separately (N=10).

Weighing pollen
When weighing low masses of pollen, static electricity can create inaccuracies on microbalances. We therefore frequently rinsed utensils with 100% ethanol, kept pollen on ice, and used anti-static guns when weighing pollen. We usually use 3 replicates of 1mg for both protein and lipid analysis per plant species, sample, or treatment.

Pollen Protein Analysis – Bradford

Materials
1. 1.7mL centrifuge tubes
2. centrifuge tube pestle
3. Vortex multitube holder
4. 0.1M NaOH - (4g/L)
5. Bio-rad Bradford assay kit, stored in refrigerator
   1. 2mg/mL bovine y-globulin protein standard
   2. Bradford reagent
6. 300uL 96-well plates
7. spectrophotometer

Standards
1. 80ug/mL protein standard
   1. add 40uL of 2mg/mL to 960uL 0.1M NaOH
2. Serial dilutions down to 1.25ug/mL
3. Add 150uL of standard to each well
4. Add 150uL Bradford reagent and mix with pipette
5. read OD in spectrophotometer at 595nm
6. determine standard curve from OD values and known concentrations

Protocol
1. Weigh 1mg samples in 1.7mL centrifuge tubes
2. Draw 750uL 0.1M NaOH into pipette
3. Add three drops 0.1M NaOH to sample
4. Grind with pestle
5. Wash pestle with remainder of NaOH into tube
6. Add another 750uL to sample = 1.5mL total
7. Vortex for 30s
8. Let sample sit overnight – go to step #5 below

<u>Alternate (most currently used)</u>:
1. Weigh 1mg pollen in 1.7mL centrifuge tubes
2. Add 1.5mL NaOH into tube
3. Votex at maximum for 10min
4. Let sample sit overnight

5. Take Bradford reagent out of refrigerator and allow to reach room temperature, turn upside down few times to mix

6. Add 100uL NaOH - to all sample wells.
7. Add 50uL sample in triplicates to well plate. This dilutes the sample concentration by 3x
8. Add 150uL Bradford reagent and mix with pipette
9. Read OD in spectrophotometer at 595nm
10. Calculate concentration based on standard curve
11. Back calculate (x3) to determine ug/mL concentration
12. Multiply by 1.5 to determine total ug protein (for 1.5mL extraction)
13. Divide by starting pollen mass to determine ug/mg protein

Pollen Lipid Analysis – based on Van Handel and Day 1988

Materials
1. 1.7mL centrifuge tubes
2. 2mL centrifuge tubes
3. 7mL Glass tubes
4. Teflon caps for glass tubes
5. Heating block at 90-110ºC
6. Centrifuge
7. Spectrophotometer
8. Large volume pipette for dispensing 5mL

Reagents
1. Sulfuric acid (95-98%)
2. Phosphoric acid (85%)
3. Vanillin
4. Sodium sulfate
5. Chloroform
6. Methanol
7. Vegetable oil

Solutions
1. 2% sodium sulfate ($NaSO_4$) solution (2g/100ml)
2. chloroform /methanol 1:1 (v/v)
   Make sure to shake well to mix solution, otherwise it will separate.
3. Vanillin-phosphoric acid reagent
   a. Dissolve 600 mg vanillin in 100 mL DI water.
   b. Add 400 mL 85% phosphoric acid.
   c. Store in the dark. Stable for several months but discard if it darkens.

Standards (use Teflon caps)
1. Lipid: 10mg veg oil (~10uL) in 10mL chloroform (1mg:1ml oil:chloroform)
   1. Add 2.5, 5, 10, 20, 50, 100, 200, 400 uL of solution to glass tube.
   2. Place rack to evaporate chloroform overnight (usually only a few hours)
   3. Add 0.2 mL of sulfuric acid
   4. Heat for 10 min at 90-110ºC
   5. Remove from heating block.
   6. Add 5ml vanillin reagent, cap, and <u>mix</u>
   7. Allow reddish color to develop; this will take approximately 5 min and will be stable up to 30 min.
   8. Determine OD at 525nm and plot µg lipid vs. OD for standard curve

Extraction of Lipid and Sugar Fractions from Pollen
1. Add ~1mg pollen to 1.5mL eppendorf centrifuge tube.
2. Add 200 uL sodium sulfate solution. Vortex
3. Wash eppendorf tube into glass tube with two x 800 uL washes of chloroform/methanol solution. Make sure everything from tube gets transferred
4. Centrifuge (2200 rcf, 5 min) – go to step #5 below

<u>Alternative:</u>
1. Add ~1mg pollen to 2mL eppendorf centrifuge tube
2. Add 200 uL sodium sulfate solution. Vortex
3. Add 1.6mL chloroform:methanol solution. Vortex
4. Centrifuge (2200 rcf, 5 min)
5. Transfer supernatant to clean glass tube (~two 800uL transfers)

6. Add 600 uL DI water to supernatant. Vortex
7. Centrifuge (2200 rcf, 5 min).
8. Discard top fraction (water/methanol) in two ~850uL transfers. This fraction can be kept for sugar analysis
9. Bottom portion (chloroform) holds the portion for lipid analysis.
10. Evaporate overnight in fume hood

Lipid Analysis

1. Wait for chloroform to completely evaporate
2. Add 200uL of sulfuric acid
3. Heat for 10 min at 100ºC (between 90-110ºC is ok but keep it consistent across all samples)
4. Remove from heating block.
5. Add 5mL vanillin reagent and mix/shake
6. Allow pink color to develop; this will take approximately 5 min and will be stable up to 30 min.
7. Add 300uL to plate in triplicates
8. Determine OD at 525 nm
9. Calculate total lipid ug from standard curve
10. Divide by starting pollen mass to determine ug/mg lipid
